# Venous thromboembolism in adrenocortical carcinoma: a retrospective analysis

**DOI:** 10.1093/oncolo/oyae099

**Published:** 2024-05-22

**Authors:** Marta Laganà, Vania Balderrama-Brondani, Kelsey Ruth Herrera, Brenda Chahla, Feyza Yaylaci, Roland L Bassett, Camilo Jimenez, Jeena Varghese, Steven G Waguespack, Matthew T Campbell, Amishi Y Shah, Cristhiam M Rojas Hernandez, Mouhammed Amir Habra

**Affiliations:** Medical Oncology Unit, Department of Medical and Surgical Specialties, Radiological Sciences and Public Health, University of Brescia at ASST Spedali Civili, Brescia, Italy; Department of Endocrine Neoplasia and Hormonal Disorders, The University of Texas MD Anderson Cancer Center, Houston, TX, United States; Department of Endocrine Neoplasia and Hormonal Disorders, The University of Texas MD Anderson Cancer Center, Houston, TX, United States; Department of Medicine, Baylor College of Medicine, Houston, TX, United States; Department of Endocrine Neoplasia and Hormonal Disorders, The University of Texas MD Anderson Cancer Center, Houston, TX, United States; Department of Endocrine Neoplasia and Hormonal Disorders, The University of Texas MD Anderson Cancer Center, Houston, TX, United States; Department of Biostatistics, The University of Texas MD Anderson Cancer Center, Houston, TX, United States; Department of Endocrine Neoplasia and Hormonal Disorders, The University of Texas MD Anderson Cancer Center, Houston, TX, United States; Department of Endocrine Neoplasia and Hormonal Disorders, The University of Texas MD Anderson Cancer Center, Houston, TX, United States; Department of Endocrine Neoplasia and Hormonal Disorders, The University of Texas MD Anderson Cancer Center, Houston, TX, United States; Department of Genitourinary Medical Oncology, The University of Texas MD Anderson Cancer Center, Houston, TX, United States; Department of Genitourinary Medical Oncology, The University of Texas MD Anderson Cancer Center, Houston, TX, United States; Section of Benign Hematology, The University of Texas MD Anderson Cancer Center, Houston, TX, United States; Department of Endocrine Neoplasia and Hormonal Disorders, The University of Texas MD Anderson Cancer Center, Houston, TX, United States

**Keywords:** adrenocortical carcinoma, prevalence, pulmonary embolism, deep venous thrombosis

## Abstract

**Background:**

Venous thromboembolism (VTE) is a leading cause of death in patients with cancer. Limited data exist about VTE in patients with adrenocortical carcinoma (ACC). The primary objective of this study was to identify the prevalence of VTE in a cohort of patients with ACC. Secondary objectives were to determine the impact of VTE events on overall survival (OS) and to describe the characteristics of VTE in patients with ACC.

**Patients and Methods:**

We retrospectively reviewed data from 289 patients with ACC cared for at a major referral center from February 2010 to June 2022.

**Results:**

VTE prevalence was 18.7% (54 events). Thirty patients (55.6%) had pulmonary embolism (PE); 12 patients (22.2%) had deep vein thrombosis (DVT); and 12 patients (22.2%) had both PE and DVT. VTE occurred after ACC diagnosis in 50 patients (92.6%) including 44 patients (88%) with stage 3 or 4 ACC. VTEs were CTCAE grade ≤2 in 32 cases (59.3%), grade 3 in 17 (31.5%), and grade 4 in 2 (3.7%). Thirteen patients (24%) died within 6 months after VTE diagnosis, although there was no statistically significant association between VTE and overall survival.

**Conclusion:**

Despite the potential to underestimate the prevalence of VTEs, we found a high frequency of VTE events in patients with ACC. A majority of VTEs occurred in the context of advanced ACC and we observed high short-term mortality. Further studies are needed to validate our findings and investigate mechanisms associated with VTE in ACC.

## Teaser

Limited data exist about venous thromboembolism (VTE) in patients with adrenocortical carcinoma. This article reports the clinical spectrum of VTE presentation in this patient population.

**Implications for Practice**

In this 13-year retrospective study, almost 1 out of 5 patients with adrenocortical carcinoma (ACC) developed venous thromboembolism (VTE) mostly after ACC diagnosis. Our study is the first to report the clinical spectrum of VTE presentation in patients with ACC including patients with metastatic disease. We found a wide variation of presentations, ranging from asymptomatic discovery to cases requiring urgent treatment for life-threatening VTE. While there was high mortality for patients with VTE in the short term, we did not find an association between VTE and long-term overall survival. The lack of association may be because of the complex and multiple factors affecting the survival of these patients, which cannot be fully captured in a retrospective analysis; further studies are warranted.

## Introduction

Adrenocortical carcinoma (ACC) is a rare cancer originating in the adrenal cortex and often associated with hypersecretion of hormones, such as glucocorticoids and sex hormones. Surgery (adrenalectomy) is the standard treatment for localized ACC. Nonetheless, ACC tends to have high recurrence rates and often requires systemic therapy.^1,2^

Venous thromboembolism (VTE) is among the leading causes of death in patients with cancer.^3^ Cancer-associated VTE has multifaceted etiology entailing patient-, cancer-, and treatment-related factors.^4^ Existing retrospective data about VTE in patients with ACC, mainly focusing on the postoperative period, are limited. In a large multicenter surgical database (2199 patients), patients with ACC who had evidence of hormonal overproduction had higher rates of developing pulmonary embolism (PE) in the immediate postoperative period compared to patients with hormonally silent ACC.^5^ Another retrospective study of 34 patients with ACC reported 8 patients (23.5%) with PE in the first 6 months after surgery; 5 were symptomatic, and 3 were incidentally discovered.^6^ More recently, a retrospective analysis of the American College of Surgeons National Surgical Quality Improvement Program database (which included 576 patients with ACC who underwent surgery) found a higher risk of postoperative VTE in patients with ACC than in patients with Cushing syndrome (CS) due to adenoma or benign adrenal diseases.^7^

We hypothesized that patients with ACC are at high risk of developing VTE given their numerous risk factors, such as tumor invasion of major vessels leading to venous stasis (inferior vena cava or renal veins), hormonal excess, abdominal surgery, and possibly cytotoxic chemotherapy. To test our hypothesis, we evaluated the prevalence of VTE events and their clinical characteristics in a large cohort of patients with ACC regardless of whether they underwent surgical intervention.

## Methods

This retrospective study was conducted at The University of Texas MD Anderson Cancer Center (MDACC). Institutional Review Board approval was obtained before starting this study (Protocol PA12-0933).

The primary objective was to identify the prevalence of VTE in a cohort of patients ≥18 years old with ACC seen at MDACC from February 2010 to June 2022. The time period was defined to ensure the accuracy of the entries and available data. Secondary objectives were to determine the impact of VTE events on overall survival (OS) and to describe the characteristics of VTE in patients with ACC.

The definition and classification of VTE events met the international consensus’s standardized reporting and analysis.^8^ Data regarding patient and tumor characteristics, clinical presentation, disease stage, surgery, and treatments were extracted from medical records. We used the European Network for the Study of Adrenal Tumors (ENS@T) staging system.^9^ The date of ACC diagnosis was defined as the date of pathological confirmation of disease, and the date of VTE was based on radiological assessment diagnostic of VTE. We documented VTE events that occurred within 3 months before ACC diagnosis or occurred after ACC diagnosis. Data regarding the VTE events were also collected, including date of diagnosis, location, symptoms at diagnosis, and the provided antithrombotic treatment. Common Terminology Criteria for Adverse Events (CTCAE) v5.0 was used to classify the VTE events. Direct tumor invasion of major vessels (tumor thrombus) was not considered a VTE event.

We assessed potential risk factors for VTE, such as smoking history (classified as current smoker, former smoker, or never smoker), surgery within 3 months before VTE diagnosis, presence of a central venous catheter, previous history or family history of VTE or mutations predisposing to hypercoagulation, oral contraceptive use, other comorbidities (obesity, organ failure, cardiopulmonary or renal disease), and Eastern Cooperative Oncology Group (ECOG) performance status.

Patients’ characteristics were collected and summarized using standard summary statistics. All the patients were analyzed and divided into 2 groups: those who developed VTE and those who did not. Two-sided *t* test or Mann-Whitney test was used to compare continuous variables, as appropriate, and chi-square was used to compare categorical variables between the groups. The Kaplan-Meier method was used to estimate the distribution of OS from the date of ACC diagnosis for each group. Patients who remained alive were censored at their last follow-up date. Variables listed in Table 1 were used in univariate analysis.

**Table 1.** Clinical and tumor characteristics of 289 patients with adrenocortical carcinoma.

Variable	Total cohort, *N* (%)	VTE, *N* (%)	No VTE, *N* (%)	*P* value
Sex				
Female	167 (57.8%)	30 (55.6%)	137 (58.3%)	0.713
Male	122 (42.2%)	24 (44.4%)	98 (41.7%)	
Age at ACC diagnosis (years), median (range)	51.7 (18-83)	53 (19.1-76.4)	50.7 (19-83.7)	0.853
Race/ethnicity				
Non-Hispanic White	232 (80.3%)	46 (85.2%)	186 (79.1%)	0.299
Hispanic	23 (8%)	3 (5.6%)	20 (8.5%)	
Black	22 (7.6%)	5 (9.3%)	17(7.2%)	
Asian	12 (4.2%)	0 (0%)	12 (5.1%)	
Hormonally active tumor				
Non-cortisol producing^a^	33 (11.4%)	7 (13%)	26 (11%)	0.772
Cortisol-producing (including mixed hormonal production)	99 (34.2%)	20 (37%)	79 (33.6%)	
Nonfunctioning	157 (54.3%)	27 (50%)	130 (55.3%)	
BMI (kg/m^2^)				
<25	85 (29.4%)	14 (25.9%)	71 (30.2%)	0.206
25 to <30	80 (27.7%)	12 (22.2%)	68 (28.95)	
≥30	102 (35.3%)	23 (42.6%)	79 (33.6%)	
NA	22 (7.6%)	5 (9.3%)	17 (7.2%)	
ENS@T stage at ACC diagnosis				
1	13 (4.5%)	0 (0%)	13 (5.5%)	0.063
2	77 (26.6%)	13 (24%)	64 (27.2%)	
3	90 (31.1%)	24 (44.4%)	66 (28%)	
4	109 (37.7%)	17 (31.5%)	92 (39.1%)	
Venous involvement of tumor (renal vein or inferior vena cava) at ACC diagnosis	53 (18.3%)	25 (46.3%)	28 (11.9%)	<0.01
Resection status				
R0	138 (47.8%)	24 (44.4%)	114 (48.5%)	0.964
R1	41 (14.2%)	7 (13%)	34 (14.5%)	
R2	5 (1.7%)	1 (1.8%)	4 (1.7%)	
RX	32 (11%)	10 (18.5%)	22 (9.4%)	
NA	73 (25.2%)	12 (22.2%)	61 (26%)	
Number of outcomes (death) at the time of this report	126 (43.6%)	26 (48.1%)	100 (42.5%)	0.455
Duration of follow-up (months), median (range)	19.5 (0.3-142.9)	25 (1.5-87.8)	17.8 (0.3-142.9)	0.112

^a^Non-cortisol–producing tumors were aldosterone-producing tumors (3 cases), estrogen-androgen–producing tumors (27 cases), aldosterone-androgen–producing tumor (2 cases), and aldosterone-estrogen-androgen–producing tumor (1 case).

Abbreviations: ACC, adrenocortical carcinoma; BMI, body mass index; ENS@T, European Network for the Study of Adrenal Tumors; VTE, venous thromboembolism; PE, pulmonary embolism; DVT, deep venous thrombosis; NA, Not available.

Statistical analysis was performed using R version 4.1.1. *P* values of <.05 were considered to indicate statistical significance. No adjustments for multiple tests were made.

## Results

### Patient characteristics

Data from 289 adult patients with ACC were reviewed and analyzed (Table 1). Fifty-four patients (18.7%) developed at least one VTE event, with a median time of appearance of 5.5 months (range − 3 to 86.4 months) relative to the date of ACC diagnosis (Table 2). Of those with VTE after ACC diagnosis (50 patients), 44 patients (88%) had ACC stage 3 or 4 disease at VTE diagnosis. In 31 (57.4%) of the 54 patients, VTE occurred within the first year of diagnosis of ACC. PE was more frequent (30 patients, 55.6%) than deep venous thrombosis (DVT; 12 patients, 22.2%); 12 patients (22.2%) had both PE and DVT (concurrent in 5 patients [9.3%]). Seven patients (13%) had more than one episode of VTE; the data presented here represent the first VTE event for each of these patients.

**Table 2.** Characteristics of the 54 patients who had VTE events.

Variable	*N* (%)
Time from initial ACC diagnosis until VTE event (months), median (range)	5.5 (−3 months to 86.4)
ECOG performance status at VTE diagnosis	
0	35 (64.8%)
1-2	15 (27.8%)
3-4	4 (7.4%)
ENS@T stage at VTE diagnosis	
VTE occurred after ACC diagnosis	50 (92.6%)
1	0 (0%)
2	3 (6%)
3	7 (14%)
4	37 (74%)
No evidence of disease	3 (6%)
VTE occurred before ACC diagnosis	4 (7.4%)
Type of VTE event (number of patients)	
PE only	30 (55.6%)
DVT only	12 (22.2%)
Concurrent VTE and DVT	5 (9.3%)
Non-concurrent PE and DVT	7 (13%)
DVT site	
Lower extremity	14 (58.3%)
Upper extremity	4 (16.7%)
NA	6 (25%)
VTE possibly related to surgery	18 (33.3%)
<3 months from surgery	9 (50%)
3-6 months from surgery	9 (50%)
Previous history of VTE	1 (1.8%)
Family history of VTE	5 (9.3%)
Smoking status at VTE diagnosis	
Never smoker	32 (59.3%)
Former smoker	20 (37%)
Every-day smoker	1 (1.8%)
NA	1 (1.8%)
VTE clinical status	
Symptomatic	19 (35.2%)
Incidental	35 (64.8%)
Adverse event grade (CTCAE v5.0)	
1	3 (5.6%)
2	29 (53.7%)
3	17 (31.5%)
4	2 (3.7%)
5	0
NA	3 (5.6%)
ACC systemic therapy at VTE diagnosis	
Mitotane	8 (14.8%)
Platinum-based chemotherapy	4 (7.4%)
Immunotherapy	3 (5.6%)
Tyrosine-kinase inhibitors	2 (3.7%)
Combination of therapies	4 (7.4%)
None	33 (61.1%)

Abbreviations: ACC, adrenocortical carcinoma; BMI, body mass index; ECOG, Eastern Cooperative Oncology Group; ENS@T, European Network for the Study of Adrenal Tumors; VTE, venous thromboembolism; PE, pulmonary embolism; DVT, deep venous thrombosis; CTCAE, Common Terminology Criteria for Adverse Events; NA: not available.

Nineteen patients (35.2%) had symptomatic VTE. According to CTCAE v5.0, 32 cases (59.3%) were grade 1 or 2, 17 (31.5%) were grade 3, and 2 (3.7%) were grade 4. Twenty-one patients (38.9%) were receiving some ACC therapy at the occurrence of VTE. Fifty (92.6%) patients received treatment for VTE: 26 (48.1%), low-molecular-weight heparins (LMWHs); 20 (37%), direct oral anticoagulants; and 4 (7.4%), vitamin K antagonists).

### Risk factors for VTE development

Regarding known risk factors for the development of VTE, 40 patients (74.1%) were older than 40 years, 9 patients (16.7%) underwent surgery within 3 months before developing VTE, 21 (38.9%) were former or current smokers, 20 (37%) had a central venous catheter, 5 (9.3%) had a family history of VTE, and 4 (7.4%) had limited mobility (ECOG performance status of 3 or 4) at the time of the VTE event. Only 1 patient (1.9%) had a previous history of VTE. Other comorbidities noted among the patients with VTE included obesity (BMI ≥ 30 kg/m^2^) in 23 patients (42.6%) and underlying cardiopulmonary compromise or renal insufficiency in 12 patients (22.2%). Twenty patients (37%) had cortisol-producing tumors.

### VTE possibly related to surgery

Eighteen patients (33.3%) had VTE within 6 months after their primary surgical resection of ACC. Of these, 11 (61.1%) had R0 resection status, 2 (11.1%) had microscopic residual disease at the primary cancer site, and 5 (27.8%) had Rx resection status. Nine patients (50%) had cortisol-producing tumors, and 8 (44.4%) had silent tumors. The median time between ACC surgery and VTE appearance after the surgery, considering only patients who developed VTE within 6 months after surgery, was 2.5 months (0-5.8 months). Nine patients (50%) developed VTE within 3 months of ACC surgery.

### Survival analysis

**Figure 1.**
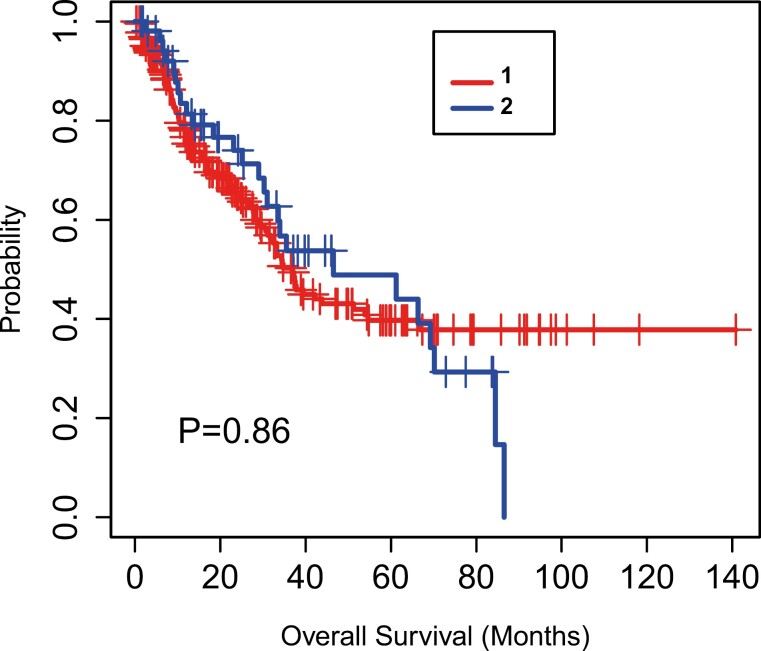
Overall survival curve. Patients were classified in 2 groups, with VTE (blue line) and with no VTE event (red line).

Twenty-six patients (48.1%) died during the follow-up period, and 13 (50%) of these patients died in the first 6 months after the VTE event. There was no statistically significant association between VTE and OS when comparing patients who did and did not develop VTE (*P* = .86; Figure 1).

## Discussion

This study establishes the high rate of VTE in a large single-center cohort of patients with ACC. Our report included patients throughout their disease course, and we did not restrict our analysis to the immediate postoperative period. Indeed, our finding of a VTE prevalence rate of 18.7% could be an underestimation of the true prevalence of VTE in patients with ACC, considering asymptomatic cases and the nature of the retrospective analysis.

The high rate of VTE in our patients with ACC is comparable to rates reported in patients with more common tumors that are known to be associated with a very high risk for thrombosis, such as pancreatic and gastric cancers, which present a prevalence of 12%-36% and 22.4% of VTE, respectively.^10-12^ Lung cancer, lymphomas, and bladder and testicular cancers are considered as high risk for VTE and present an incidence rate of VTE varying between 5.6% and 16.9%.^13-15^ The increased risk of VTE in patients with ACC in our cohort compared with most other cancers could be explained by abdominal surgery, ACC systemic treatment, metastatic disease, or a hypercoagulable state due to the cancer itself or to hormonal production by the ACC.^6,16^ Our cohort was heterogeneous regarding the potential and classic risk factors for VTE, and only 21 cases (38.9%) had at least one risk factor present. Certain cancers (lung, uterus, bladder, pancreas, stomach, and kidney) when metastatic are associated with VTE incidence rate at 1-year that ranges from 4 to 13 times higher than patients with localized disease.^17^ Forty-four VTEs (81.5%) were diagnosed in patients with advanced (stage 3 and 4) ACC, which could point toward the hypercoagulable state due to aggressive cancer. Cortisol hypersecretion is a well-known hypercoagulability factor.^18,19^ Isand et al^20^ published a cohort with 2174 patients with CS and reported a prevalence of 4.4% (95 cases) of VTE, and sex (male), high urinary cortisol levels, and repeated surgeries were associated with higher risk for VTE. Interestingly, sex and hypercortisolemia was not associated with the development of VTE events in our cohort and cortisol secretion did not impact the overall survival in patients with VTE.

Venous involvement at ACC diagnosis was the only variable with statistical significance between the groups in our cohort. It remains unclear if ACC produces any cancer-specific substances that could have a role in thrombogenesis, as seen in adenocarcinomas of the pancreas, lung, and gastrointestinal tract.^21^

In a Danish population-based study (499 092 patients), diagnosis of VTE in patients with cancer within the 6 months before the cancer diagnosis was more frequent than in individuals without cancer from the general population who were tracked for the same period (hazard ratio [HR], 6; 95% CI, 5.7-6.3).^22^ Also, there was an increased incidence rate of VTE until 12 months after cancer diagnosis, with HRs varying according to cancer type from 2.5 to 41.7.^22^ In our patients, 37 patients (68.5%) had VTE events within 3 months before ACC diagnosis on up to 12 months after diagnosis.

About half of VTEs in patients with cancer are incidentally diagnosed cases, with lung and pancreas cancer cohorts presenting the highest rates of incidental VTEs.^23-25^ It is not clear whether patients with incidental VTEs have higher rates of morbidity or mortality when compared with patients with symptomatic VTEs; however, treatment is highly recommended for incidental cases.^23,26^ We found 35 patients (64.8%) with incidental VTEs and did not find differences between symptomatic and incidental cases. We did not notice a temporal increase in VTE cases, as was described by Mulder et al^26^; this difference could be the result of our established ACC management protocol with imaging studies and systemic therapies.

The American Society of Clinical Oncology recommends pharmacological thromboprophylaxis for patients who undergo major cancer surgery, since they have a high risk for VTEs.^27^ Until further data become available, we still follow our current institutional VTE prophylaxis for adult patients admitted to hospital for abdominal/pelvic surgery for cancer. If the patient does not have a contraindication to pharmacological prophylaxis (ie, active bleeding, thrombocytopenia, recent CNS bleed or neurosurgery, severe uncontrolled malignant hypertension), we use LMWH or heparin and sequential compression device for at least 7-10 days, with an extension to a total of 28 days of prophylaxis for VTE high-risk patients. Although our patients received thromboprophylaxis in this scenario, 18 (33.3%) of our cases occurred after surgery, and 50% of them occurred in the first 3 months after adrenalectomy, with a median of 2.5 (0-5.8) months. Of these 18 cases, 13 (72.2%) were PEs. Durmusoglu et al^6^ published a VTE incidence rate of 23.5% in their surgical ACC cohort (34 patients), and all 8 cases were PEs. Seven (87.5%) of these VTEs occurred during the first 3 months after surgery, compared with 9 of our patients with VTE (50%) in the same time frame.^6^ Durmusoglu et al^6^ reported that 75% of patients with VTE had cortisol-producing tumors, and 25% had silent tumors. In comparison, we reported 36.6% of VTE cases with cortisol-producing tumors and 50% with silent tumors in our cohort. The frequency of advanced ACC cases was similar in both cohorts, although we found a higher frequency of metastatic disease (stage 4; 74% vs 50%). There is no specific recommendation regarding the duration of thromboprophylaxis after surgery in patients with ACC.^16,27^ Moreover, the current VTE risk scores do not consider ACC as an intermediate- or high-VTE-risk cancer.^28,29^ We suggest that thromboprophylaxis for patients with ACC should be warranted in case of major abdominopelvic surgery for 28 days according to guidelines.^27^

The risk of thromboembolism in patients with cancer is higher than in individuals without cancer and is a major health problem and a leading cause of mortality after the cancer itself.^3,16^ In the Danish population-based study, the mortality rates of patients with cancer with VTE were 2- to 3-fold higher than rates for individuals without VTE.^22^ Moreover, VTEs can impact morbidity rates, interrupting cancer treatment and decreasing quality of life.^3,22,30^ We did not find an association of VTE with OS compared with our control group (patients with ACC who did not have VTE events). One explanation is that these patients were treated at a tertiary center and received an early VTE diagnosis with prompt treatment, which is likely to improve outcomes.^31^ However, 50% of the deaths in our VTE cohort occurred within 6 months after the VTE event; this points to an impact of VTE on morbidity in these advanced patients with ACC.

## Conclusion

We found a high frequency of VTE events in patients with ACC, with varied presentation and severity, and the majority occurring in advanced ACC. We observed a high short-term mortality after VTEs. Further prospective and multicenter studies assessing risk scores^28,29^ for VTE should be performed to determine their applicability in ACC cohorts and possibly guide personalized thromboprophylaxis for VTE high-risk patients with advanced ACC.

## Data Availability

The data underlying this article will be shared on reasonable request to the corresponding author.

## References

[1] Daher M , Varghese J, Gruschkus SK, et al. Temporal trends in outcomes in patients with adrenocortical carcinoma: a multidisciplinary referral-center experience. J Clin Endocrinol Metab. 2022;107(5):1239-1246. 10.1210/clinem/dgac046 35092681 PMC9016449

[2] Fassnacht M , Terzolo M, Allolio B, et al.; FIRM-ACT Study Group. Combination chemotherapy in advanced adrenocortical carcinoma. N Engl J Med. 2012;366(23):2189-2197. 10.1056/NEJMoa1200966 22551107

[3] Khorana AA , Francis CW, Culakova E, Kuderer NM, Lyman GH. Thromboembolism is a leading cause of death in cancer patients receiving outpatient chemotherapy. J Thromb Haemost. 2007;5(3):632-634. 10.1111/j.1538-7836.2007.02374.x 17319909

[4] Falanga A , Marchetti M. Cancer-associated thrombosis: enhanced awareness and pathophysiologic complexity. J Thromb Haemost. 2023;21(6):1397-1408. 10.1016/j.jtha.2023.02.029 36931602

[5] Parikh PP , Rubio GA, Farra JC, Lew JI. Nationwide analysis of adrenocortical carcinoma reveals higher perioperative morbidity in functional tumors. Am J Surg. 2018;216(2):293-298. 10.1016/j.amjsurg.2017.08.018 28859919

[6] Durmusoglu J , Timmers H, van Houten P, et al. Venous thromboembolism in patients with adrenocortical carcinoma after surgery. Endocr Connect. 2020;9(9):874-881. 32784266 10.1530/EC-20-0299 PMC7487187

[7] Moore MD , Agrusa C, Ullmann TM, et al. Risk factors for venous thromboembolism (VTE) after adrenalectomy for adrenal cortical neoplasms. J Surg Oncol. 2022;126(7):1176-1182. 10.1002/jso.27059 35997946

[8] Carrier M , Khorana AA, Zwicker JI, et al.; Subcommittee on Haemostasis and Malignancy for the SSC of the ISTH. Venous thromboembolism in cancer clinical trials: recommendation for standardized reporting and analysis. J Thromb Haemost. 2012;10(12):2599-2601. 10.1111/jth.12028 23362524

[9] Fassnacht M , Johanssen S, Quinkler M, et al.; German Adrenocortical Carcinoma Registry Group. Limited prognostic value of the 2004 International Union Against Cancer staging classification for adrenocortical carcinoma: proposal for a Revised TNM Classification. Cancer. 2009;115(2):243-250. 10.1002/cncr.24030 19025987

[10] Abdel-Razeq H , Mustafa R, Sharaf B, et al. Patterns and predictors of thromboembolic events among patients with gastric cancer. Sci Rep. 2020;10(1):18516. 10.1038/s41598-020-75719-w 33116272 PMC7595162

[11] Yuan S , Sun Y, Chen J, Li X, Larsson SC. Long-term risk of venous thromboembolism among patients with gastrointestinal non-neoplastic and neoplastic diseases: a prospective cohort study of 484 211 individuals. Am J Hematol. 2023;99(2):172-181. 10.1002/ajh.27106 37753710

[12] Campello E , Ilich A, Simioni P, Key NS. The relationship between pancreatic cancer and hypercoagulability: a comprehensive review on epidemiological and biological issues. Br J Cancer. 2019;121(5):359-371. 10.1038/s41416-019-0510-x 31327867 PMC6738049

[13] Mulder FI , Candeloro M, Kamphuisen PW, et al.; CAT-prediction collaborators. The Khorana score for prediction of venous thromboembolism in cancer patients: a systematic review and meta-analysis. Haematologica. 2019;104(6):1277-1287. 10.3324/haematol.2018.209114 30606788 PMC6545838

[14] Hohaus S , Bartolomei F, Cuccaro A, et al. Venous thromboembolism in lymphoma: risk stratification and antithrombotic prophylaxis. Cancers (Basel). 2020;12(5):1291. 10.3390/cancers12051291 32443753 PMC7281118

[15] Caruso V , Di Castelnuovo A, Meschengieser S, et al. Thrombotic complications in adult patients with lymphoma: a meta-analysis of 29 independent cohorts including 18 018 patients and 1149 events. Blood. 2010;115(26):5322-5328. 10.1182/blood-2010-01-258624 20378755

[16] Falanga A , Ay C, Di Nisio M, et al.; ESMO Guidelines Committee. Venous thromboembolism in cancer patients: ESMO Clinical Practice Guideline. Ann Oncol. 2023;34(5):452-467. 10.1016/j.annonc.2022.12.014 36638869

[17] Chew HK , Wun T, Harvey D, Zhou H, White RH. Incidence of venous thromboembolism and its effect on survival among patients with common cancers. Arch Intern Med. 2006;166(4):458-464. 10.1001/archinte.166.4.458 16505267

[18] Wagner J , Langlois F, Lim DST, McCartney S, Fleseriu M. Hypercoagulability and risk of venous thromboembolic events in endogenous Cushing’s syndrome: a systematic meta-analysis. Front Endocrinol (Lausanne). 2019;9:805. 10.3389/fendo.2018.00805 30745894 PMC6360168

[19] Kastelan D , Dusek T, Kraljevic I, et al. Hypercoagulability in Cushing’s syndrome: the role of specific haemostatic and fibrinolytic markers. Endocrine. 2009;36(1):70-74. 10.1007/s12020-009-9186-y 19381886

[20] Isand K , Feelders R, Brue T, et al.; Ercusyn Study Group. High prevalence of venous thrombotic events in Cushing’s syndrome: data from ERCUSYN and details in relation to surgery. Eur J Endocrinol. 2024;190(1):75-85. 10.1093/ejendo/lvad176 38146835

[21] Khorana AA , Connolly GC. Assessing risk of venous thromboembolism in the patient with cancer. J Clin Oncol. 2009;27(29):4839-4847. 10.1200/JCO.2009.22.3271 19720906 PMC2764392

[22] Mulder FI , Horvath-Puho E, van Es N, et al. Venous thromboembolism in cancer patients: a population-based cohort study. Blood. 2021;137(14):1959-1969. 10.1182/blood.2020007338 33171494

[23] Di Nisio M , Carrier M. Incidental venous thromboembolism: is anticoagulation indicated? Hematology Am Soc Hematol Educ Program. 2017;2017(1):121-127. 10.1182/asheducation-2017.1.121 29222246 PMC6142551

[24] Menapace LA , Peterson DR, Berry A, Sousou T, Khorana AA. Symptomatic and incidental thromboembolism are both associated with mortality in pancreatic cancer. Thromb Haemost. 2011;106(2):371-378. 10.1160/TH10-12-0789 21713322

[25] Tiseo M , Bersanelli M, Pesenti Barili M, et al. Asymptomatic pulmonary embolism in lung cancer: prevalence and analysis of clinical and radiological characteristics in 141 outpatients. Tumori. 2012;98(5):594-600. 10.1177/030089161209800509 23235754

[26] Mulder FI , Di Nisio M, Ay C, et al. Clinical implications of incidental venous thromboembolism in cancer patients. Eur Respir J. 2020;55(2):1901697. 10.1183/13993003.01697-2019 31727694

[27] Key NS , Khorana AA, Kuderer NM, et al. Venous thromboembolism prophylaxis and treatment in patients with cancer: ASCO clinical practice guideline update. J Clin Oncol. 2020;38(5):496-520. 10.1200/JCO.19.01461 31381464

[28] Khorana AA , Kuderer NM, Culakova E, Lyman GH, Francis CW. Development and validation of a predictive model for chemotherapy-associated thrombosis. Blood. 2008;111(10):4902-4907. 10.1182/blood-2007-10-116327 18216292 PMC2384124

[29] Li A , De Las Pozas G, Andersen CR, et al. External validation of a novel electronic risk score for cancer-associated thrombosis in a comprehensive cancer center. Am J Hematol. 2023;98(7):1052-1057. 10.1002/ajh.26928 37067102 PMC10330124

[30] Lloyd AJ , Dewilde S, Noble S, Reimer E, Lee AYY. What impact does venous thromboembolism and bleeding have on cancer patients’ quality of life? Value Health. 2018;21(4):449-455. 10.1016/j.jval.2017.09.015 29680102

[31] Canonico ME , Santoro C, Avvedimento M, et al. Venous thromboembolism and cancer: a comprehensive review from pathophysiology to novel treatment. Biomolecules. 2022;12(2):259. 10.3390/biom12020259 35204760 PMC8961522

